# Graphoepitaxial Control of Lamellar Block Copolymer Alignment in Wide Trenches: Effects of Trench Width and Sidewall Tilt

**DOI:** 10.3390/polym18050557

**Published:** 2026-02-25

**Authors:** June Huh

**Affiliations:** Department of Chemical and Biological Engineering, Korea University, Seoul 02841, Republic of Korea; junehuh@korea.ac.kr

**Keywords:** block copolymer, directed self-assembly, graphoepitaxy, lamellae, alignment, kinetic trapping, trench width, sidewall tilt (taper)

## Abstract

Graphoepitaxy provides a robust route to align lamellar block copolymers in topographic trenches, yet alignment often degrades rapidly as the trench width increases into the wide-trench regime. Here, mesoscale density functional simulations under thermal annealing are used to quantify width- and geometry-dependent ordering of symmetric lamella-forming block copolymers confined in trenches. A Fourier-based alignment metric reveals a sharp, sigmoidal decay of alignment with trench width normalized by the natural lamellar period, indicating a crossover between (i) a globally aligned state established by wall-guided propagation and (ii) a misoriented, kinetically trapped state produced by bulk-like interior nucleation followed by domain impingement. This width dependence is well captured by a logistic form, yielding a characteristic crossover width and transition sharpness that compactly describe the accessible alignment window. Parameter sweeps show that increasing incompatibility shifts the crossover to smaller widths, whereas stronger sidewall surface fields extend the accessible width range with diminishing returns at large fields; in the range examined, film thickness has little influence on the crossover. Finally, simulations in trapezoidal trenches demonstrate that high alignment persists for moderate sidewall taper, while larger taper promotes lamellar bending and defects. A geometric criterion based on the variation in trench width across the film thickness, using a numerical threshold derived from strong-segregation theory, rationalizes the observed onset of degradation when this variation approaches approximately 1.4 lamellar periods. These results provide a mechanistic framework and quantitative guidelines for extending graphoepitaxial lamellar alignment beyond the narrow-confinement regime.

## 1. Introduction

Block copolymer (BCP) directed self-assembly (DSA) has emerged as a powerful bottom-up patterning strategy that complements advanced lithographic techniques in nanoscale manufacturing [1,2,3,4]. By exploiting the intrinsic periodicity and thermodynamic driving forces of BCP microphase separation, DSA enables the formation of highly regular line–space and contact-hole patterns that are difficult to realize by top-down lithography alone. Among various DSA approaches, graphoepitaxy—in which topographic features such as trenches or grooves guide self-assembly—has been widely explored as a robust and process-tolerant route for directing BCP ordering in technologically relevant geometries.

In graphoepitaxial DSA, the sidewalls of the guiding template can nucleate and orient ordered domains, and this ordering may propagate inward to impose registration over the entire template region [2,5]. Such sidewall-guided ordering is particularly effective for lamella-forming BCPs, where preferential interactions at the trench walls and geometric confinement break orientational degeneracy and promote trench-parallel lamellae with low defect density [6]. Recent advances have further refined graphoepitaxial process windows and integration schemes, underscoring the continued relevance of this approach in advanced patterning flows [7,8,9,10,11]. Historically, graphoepitaxial alignment of lamella-forming BCPs in topographic trenches was established by leveraging interfacial boundary conditions (e.g., a neutral bottom surface combined with preferential sidewalls) and geometric commensurability, which together break the in-plane orientational degeneracy and promote trench-parallel lamellae within a finite process window (e.g., [12,13,14]). Subsequent advances improved defect control and process latitude through template and integration innovations, including disposable/soft confinement schemes and optimized topographies such as shallow or tapered trenches, and extended graphoepitaxy to more complex, circuit-relevant or curved motifs (e.g., [15,16,17]). In parallel, theoretical and computational studies—ranging from self-consistent field theory (SCFT) to mesoscale phase-field/coarse-grained simulations—have been widely used to map commensurability windows, quantify surface-interaction requirements, and analyze kinetic trapping and defect pathways in directed self-assembly (e.g., [18,19,20]). Motivated by these developments, the present work focuses on the wide-trench regime (typically W/λ≳O(10)) where alignment commonly breaks down and develops a kinetic-competition description linking wall-guided ordering fronts and bulk-like interior nucleation.

Beyond nanoelectronics, the ability of BCP-based self-assembly to generate dense, periodic nanopatterns over large areas has stimulated growing interest in nanophotonic and optical applications. In these contexts, aligned line/space motifs serve as essential building blocks for resonant gratings, metasurfaces, and polarization-control elements, where wafer-scale alignment and long-range order are critical for optical performance [21,22]. Importantly, device-level layouts, sparse templating strategies, and integration constraints in such applications naturally introduce micron-scale open regions or wide guiding features [2,23], in which the sidewalls must impose orientational order over distances far exceeding a few BCP periods.

Despite substantial progress under narrow confinement, a critical and practically important limitation remains insufficiently understood: the rapid degradation of lamellar alignment as trench width increases. From a manufacturing standpoint, extending graphoepitaxial control to wider trenches would relax template density requirements, improve pattern integration flexibility, and reduce reliance on extremely dense pre-patterning. However, both experimental and computational studies consistently show that beyond a characteristic width, well-aligned lamellae are replaced by polycrystalline or defect-rich morphologies, even under otherwise favorable surface conditions and thermal annealing protocols [14,15,24]. While nonthermal approaches such as solvent-vapor annealing can enhance chain mobility, they introduce additional process variables—including swelling, solvent uptake kinetics, and drying history—that complicate integration and reproducibility [25,26,27,28]. These considerations motivate the need for a predictive, kinetics-based understanding of why alignment breaks down in wide trenches under process-relevant thermal conditions.

Motivated by these considerations, the present work focuses on the wide-trench regime (typically W/λ≳O(10), where *W* is the trench width and λ is the natural lamellar period), where alignment commonly breaks down, and develops a kinetic-competition description linking wall-guided ordering fronts and bulk-like interior nucleation. Using mesoscale density functional simulations under thermal annealing conditions, we systematically examine how lamellar ordering evolves as the trench width increases from sub-micron to micrometer scales. Rather than treating alignment as a binary outcome, we analyze the kinetic competition between wall-initiated ordering fronts and bulk-like nucleation in the trench interior. We show that the breakdown of alignment in wide trenches reflects an intrinsic kinetic limitation—arising from this competition—rather than simply insufficient wall affinity. This framework yields a compact crossover metric that quantifies the characteristic width beyond which wall-guided alignment becomes kinetically inaccessible, and it clarifies how molecular incompatibility, film thickness, and sidewall selectivity shift this width scale.

In addition, whereas most idealized analyses assume perfectly vertical sidewalls, practical etched trenches commonly exhibit finite sidewall taper. Such trapezoidal cross-sections modify the effective confinement and the geometry of ordering fronts, and may therefore perturb the width-driven alignment window. We therefore examine sidewall tilt both as an experimentally unavoidable non-ideality and as a potential geometric control parameter for improving alignment robustness without altering polymer chemistry or surface treatments. This framework yields a predictive design strategy for wide-trench graphoepitaxy, offering quantitative guidelines for achieving highly aligned lamellae under process-relevant geometries.

## 2. Simulation Methods

We investigate the ordering of lamella-forming block copolymers (BCPs) confined in graphoepitaxial trenches by employing mesoscale density functional theory simulations. Microphase separation between the A and B blocks is represented by an order parameter Ψ(r), defined as the deviation of the local A-monomer volume fraction ϕ(r) from its spatial mean *f*, i.e., Ψ(r)=ϕ(r)−f. The time evolution of Ψ(r) is described by a conserved Cahn–Hilliard-type diffusion dynamics [29,30,31]:

(1) $$\frac{\partial \Psi (r)}{\partial t}=M\nabla^{2}\frac{\partial (F+F_{\mathrm{surf}})}{\partial \Psi (r)}+\xi \left(r\right),$$

where *M* is the mobility (set to unity in this study), *F* denotes the bulk free-energy functional of the BCP, $F_{\mathrm{surf}}$ accounts for preferential interactions with confining interfaces, and ξ(r) represents thermal noise. In the present work, however, we do not apply an additional time-dependent noise during the evolution (i.e., ξ=0 in practice). Instead, stochasticity is introduced through the weakly disordered initial condition, Ψ(r,0)∼u(−η,η) (uniform distribution), with η=0.1 unless otherwise noted. All reported alignment data are obtained by averaging over multiple independent runs with different initial realizations.

The bulk contribution *F* is modeled using a Landau–Ginzburg expansion for diblock copolymers, combined with a nonlocal term that suppresses long-wavelength composition inhomogeneity and selects a characteristic lamellar periodicity:

(2) $$\begin{array}{cc} F(\Psi ) & =\int dr\left[-\frac{\tau }{2}\Psi^{2}\left(r\right)+\frac{\mu }{3!}\Psi^{3}\left(r\right)+\frac{\lambda }{4!}\Psi^{4}\left(r\right)+\frac{D}{2}{\left\{\nabla \Psi (r)\right\}}^{2}\right]\\ \; & \;+\frac{b}{2}\int dr_{1}\int dr_{2}G\left(r_{1}-r_{2}\right)\Psi \left(r_{1}\right)\Psi \left(r_{2}\right), \end{array}$$

where τ plays the role of a temperature-like control parameter related to the Flory–Huggins interaction parameter χ, and μ, λ, *D*, and *b* are coefficients determined by the diblock architecture. The Green-function term introduces a long-range penalty that disfavors macroscopic segregation and stabilizes periodic lamellar ordering.

For a diblock copolymer, the molecular parameters are approximated as

(3) $$\begin{array}{cc} \; & \tau =2\left(\chi -\chi_{s}\right)+\frac{\sqrt{3}}{Nf^{3/2}{\left(1-f\right)}^{3/2}},\;\mu =\Gamma_{3}/N,\;\lambda =\Gamma_{4}\left(0,0\right)/N,\\ \; & D=\frac{1}{12f(1-f)},\;b=\frac{9}{N^{2}f^{2}{\left(1-f\right)}^{2}}, \end{array}$$

where $\chi_{s}$ is the spinodal value of χ, *N* is the degree of polymerization, and $\Gamma_{3}$ and $\Gamma_{4}\left(0,0\right)$ are Leibler vertex functions [32,33,34].

Preferential wetting and surface selectivity are incorporated via a linear surface free-energy term,

(4) $$F_{\mathrm{surf}}=\sum_{\alpha }\int dr\;s_{\alpha }\left(r\right)\;\Psi \left(r\right),$$

in which $s_{\alpha }\left(r\right)$ denotes the surface field acting on Ψ(r) at locations in contact with surface α. In the trench geometry considered here, α=w, *s*, and *t* correspond to the trench sidewalls, substrate, and free surface, respectively. The magnitude and sign of $s_{\alpha }$ reflect the imbalance in interfacial tensions between surface α and the two blocks. The surface field $s_{\alpha }$ in Equation (4) represents preferential wetting at interface α (α=w,s,t for sidewalls, substrate, and free surface). $s_{\alpha }=0$ corresponds to a neutral surface, while $|s_{\alpha }|$ controls the strength of selectivity; the sign determines which block preferentially wets the interface (for a symmetric diblock, reversing the sign is equivalent to relabeling the blocks). Unless stated otherwise, we use a moderately selective sidewall field $s_{w}=0.5$ and neutral substrate/top surface fields $s_{s}=s_{t}=0$.

The governing diffusion equation is solved numerically on a discrete lattice using an explicit Cell Dynamics Simulation (CDS) scheme (after Oono and Puri) [35,36]. Each simulation is initialized from a weakly disordered state by assigning random values to the order parameter at every grid point, Ψ(r)∼u(−η,η), where u(−η,η) is a uniform distribution and η=0.1. Thermal annealing is modeled as time evolution at fixed χN starting from this weakly disordered initial condition, corresponding to a nearly homogeneous melt with small composition fluctuations at t=0. Solid boundaries are treated with no-flux (Neumann) conditions, ${\left.\frac{\partial \Psi }{\partial n}\right|}_{s}=0$, implying zero normal flux (reflecting boundaries). Periodic boundary conditions are imposed only along the trench direction, while confinement is enforced by the trench sidewalls. Reflective boundary conditions are applied in the *z* direction and at all trench surfaces.

The computational domain uses $L_{x}\times L_{y}\times L_{z}$ grid points, where $L_{x}=W$ spans the trench-width direction, $L_{y}$ the trench (long) direction, and $L_{z}=h$ the thickness direction (all in grid units). Unless otherwise noted, we choose the trench length such that the normalized length is fixed at ${\tilde{L}}_{y}\equiv L_{y}/\lambda =30$, where λ is the natural lamellar period for the same parameter set. Accordingly, $L_{y}$ is set to $L_{y}\approx 30\lambda$ (rounded to the nearest integer grid point). The model system corresponds to a symmetric lamella-forming BCP with f=0.5 and χN=20. The natural lamellar period λ is tuned by varying *N*. At fixed χN, the degree of polymerization *N* enters the mesoscale model through the coefficient mapping in Equation (3), and it also sets the intrinsic period λ selected by the competition between the gradient and nonlocal penalty terms. Accordingly, we report results using normalized geometry $\tilde{W}=W/\lambda$, $\tilde{h}=h/\lambda$, and ${\tilde{L}}_{y}=L_{y}/\lambda$. To assess the residual sensitivity to *N* beyond this normalization, we performed additional simulations at *N* = 200 and 300 (with χN fixed) under the same normalized geometry; the results are summarized in Figure S1. In this range (N=100–300) at fixed χN and under identical normalized geometry, we do not observe a systematic change in the late-time ordering outcome.

Trench geometries are parameterized by the trench width *W*, film thickness *h*, and sidewall angle θ. To mimic experimentally realistic trench openings, the guiding template includes an upper capping/rounding region near the trench mouth (see cross-sectional snapshots). The BCP film is assumed to fill the trench cavity up to the flat opening plane at which the capping begins, so that the free surface remains planar; accordingly, the film thickness *h* corresponds to the effective trench depth (i.e., the sidewall height in contact with the polymer). The angle θ is varied systematically to assess the effect of wall taper on ordering behavior. Unless stated otherwise, the sidewalls are taken to be selective ($s_{w}\neq 0$), and the substrate and top surface are assumed to be neutral ($s_{s}=s_{t}=0$).

For numerical stability of the explicit CDS update, the time step is chosen to satisfy

(5) $$\begin{array}{c} \Delta t<\frac{\Delta r^{4}}{18M-3\left(A-1\right)\Delta r^{2}}, \end{array}$$

where Δt is the time increment, Δr is the grid spacing, *M* is the mobility in the evolution equation, and *A* is related to τ (approximately A≈1+τ). With Δr fixed, M=1, and *A* in the range 1.1–1.25, the stability criterion yields Δt<0.056. All simulations were performed using self-developed CDS code with Δt=0.05.

## 3. Results

**Figure 1:**
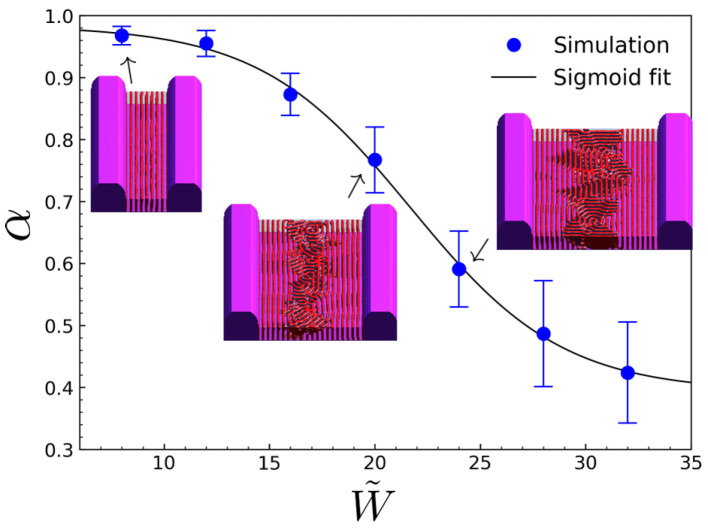
Width-dependent decay of lamellar alignment in graphoepitaxial trenches for a representative simulation condition (χN=18 with N=100, normalized film thickness $\tilde{h}=h/\lambda =4$, sidewall surface field $s_{w}=0.5$, and neutral surface fields $s_{s}=0.0$ and $s_{t}=0.0$). The alignment degree α is plotted as a function of the normalized trench width $\tilde{W}=W/\lambda$, where λ is the bulk lamellar period. Symbols denote simulation results and the solid line is a sigmoidal fit highlighting a crossover from wall-dominated ordering to interior-dominated morphologies. Representative morphologies at selected $\tilde{W}$ values illustrate the evolution from coherent wall-guided lamellae to maze-like interior domains with residual sidewall ordering.

Figure 1 summarizes the dependence of lamellar alignment on the trench width normalized by the bulk lamellar period ($\tilde{W}=W/\lambda$) for a representative simulation condition (χN=20 with N=100, normalized film thickness $\tilde{h}=h/\lambda =4$, sidewall surface field $s_{w}=0.5$, and neutral surface fields for the substrate and film top, $s_{s}=0.0$ and $s_{t}=0.0$). The lamellar alignment is quantified using a Fourier-based metric defined as

(6) $$\alpha =\frac{I_{\|}}{I_{\|}+I_{\mathrm{np}}}=\frac{I_{\|}}{I_{\mathrm{tot}}}.$$

Here $I\left(q\right)=|\tilde{\Psi }{\left(q\right)|}^{2}$ denotes the power spectrum of the order-parameter field, and the wave vector q is expressed in polar coordinates (q,φ), where φ=0 corresponds to wave vectors parallel to the trench sidewalls. The spectral weights are evaluated by integrating I(q,φ) over the first-order annulus $q\in [q_{\min},q_{\max}]$ centered at $q^{*}≃2\pi /\lambda$:

(7) $$\begin{array}{cc} \;\;\;\;\;\;I_{\|}= & \int_{q_{\min}}^{q_{\max}}dq\int_{-\Delta \varphi }^{+\Delta \varphi }d\varphi \;I\left(q,\varphi \right), \end{array}$$

(8) $$\begin{array}{cc} \;\;\;\;\;\;I_{\mathrm{tot}}= & \int_{q_{\min}}^{q_{\max}}dq\int_{0}^{2\pi }d\varphi \;I\left(q,\varphi \right), \end{array}$$

(9) $$\begin{array}{cc} I_{\mathrm{np}}= & I_{\mathrm{tot}}-I_{\|}, \end{array}$$

with Δφ=π/12 unless otherwise noted. Here $\left[q_{min},q_{max}\right]$ denotes the radial interval used to isolate the first-order lamellar peak centered at $q^{*}$; in this work, we select this interval to encompass the primary peak in I(q), and the results are insensitive to moderate variations of this range. To assess statistical variability arising from stochastic initial conditions (random initial disorder Ψ(r,0)∼u(−η,η)), all alignment data were obtained by averaging over multiple independent simulation runs for each parameter set. Unless otherwise noted, each data point represents the mean alignment degree α computed from 10 simulations initialized with different random initial conditions. Error bars shown in the figures indicate the corresponding standard deviation, reflecting run-to-run variability rather than fitting uncertainty.

For narrow trenches ($\tilde{W}≲{\tilde{W}}_{0}$), a single dominant lamellar orientation spanning the entire trench is obtained, yielding alignment degrees close to unity. As $\tilde{W}$ increases, however, the alignment degree decreases sharply over a relatively narrow width range and approaches a low but finite value, consistent with the representative morphologies in Figure 1 that evolve from coherent wall-guided lamellae to maze-like interior domains with residual sidewall ordering. The sigmoidal decay of $\alpha (\tilde{W})$ indicates that the loss of alignment reflects a crossover between two competing kinetic pathways rather than a gradual accumulation of small distortions. Specifically, ordering can proceed through (i) a wall-guided pathway, in which lamellae nucleated at the trench sidewalls propagate across the entire width to yield a globally aligned state, and (ii) a bulk-like pathway, in which independent nucleation occurs in the trench interior, followed by collisions between domains of different orientations and subsequent kinetic trapping.

**Figure 2:**
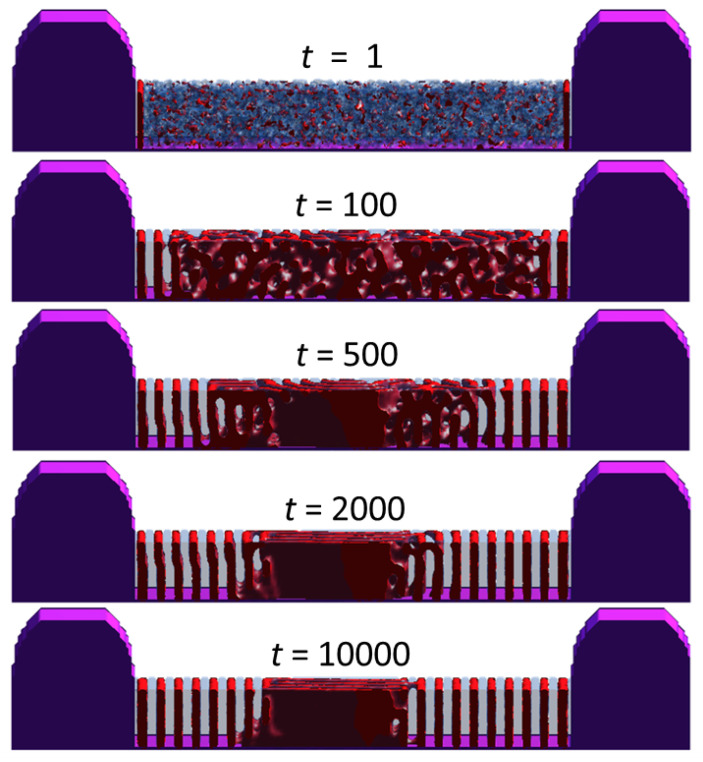
Time evolution of lamellar ordering in a representative wide trench with normalized width $\tilde{W}=28$ under the representative simulation condition of Figure 1 (χN=20, $\tilde{h}=4$, $s_{w}=0.5$, $s_{s}=0.0$ and $s_{t}=0.0$). Snapshots are shown at t=1, 100, 500, 2000, and 10,000 (arbitrary simulation units).

Figure 2 visualizes this kinetic competition through the time evolution of a representative wide-trench morphology at $\tilde{W}=28$ under the baseline condition of Figure 1. Because the sidewall surface field provides a deterministic bias, wall-induced plating appears at very early times adjacent to the trench sidewalls, producing locally layered lamellae that seed the wall-guided orientation; the wall-guided propagation corresponds to the inward advance of this wall-seeded ordered region as an ordering front. In contrast, the first bulk-like interior nucleus becomes morphologically identifiable only after a finite induction time, which we interpret as a stochastic waiting time for interior nucleation, $\tau_{\mathrm{bulk}}$, in contrast to the deterministic wall-guided propagation time, $\tau_{\mathrm{wall}}$. Therefore, whether interior nucleation occurs before or after the wall-guided front spans the trench is governed by the competition between these two timescales: if the wall-guided front spans the width before a stable interior nucleus emerges, a globally aligned state results; otherwise, an interior nucleus forms first and subsequent domain impingement leads to kinetic trapping.

In wide trenches, however, independent bulk-like nucleation events arise in the trench interior before the wall-guided front spans the full width. These interior nuclei develop lamellar domains that are generally misoriented relative to the wall-guided lamellae and grow toward the trench center. When the wall-guided and interior-grown domains impinge, a persistent grain boundary forms between the competing orientations. Subsequent evolution is limited to weak local adjustments of this boundary, indicating kinetic trapping into a long-lived, metastable morphology. Consequently, alignment improves during the wall-front propagation stage but saturates once domain impingement and kinetic trapping set in. To confirm that the wide-trench morphology is kinetically trapped rather than slowly coarsening toward a globally aligned state, we extended the representative $\tilde{W}=28$ simulation to substantially longer times and found that the alignment metric α(t) reaches a plateau after $t\approx 3\times 10^{4}$ with no subsequent systematic drift. These observations indicate that, for a given trench width, the final alignment state is not set by a gradual degradation of order, but rather by which of the two competing kinetic pathways ultimately prevails. The system therefore evolves toward either a globally aligned state established by wall-guided propagation or a partially aligned, kinetically trapped state resulting from interior nucleation and subsequent domain collision.

Within this kinetic picture, the measured alignment degree can be interpreted as an average over these two limiting outcomes. Let $\alpha_{\mathrm{good}}\approx 1$ denote the alignment degree of a globally aligned state, and $\alpha_{0}$ the limiting alignment degree attained in wide trenches. If $P_{\mathrm{good}}\left(\tilde{W}\right)$ denotes the probability that the wall-guided pathway dominates at a given normalized trench width, the observed alignment degree may be written as

(10) $$\alpha \left(\tilde{W}\right)=\alpha_{0}+\left(\alpha_{\mathrm{good}}-\alpha_{0}\right)P_{\mathrm{good}}\left(\tilde{W}\right).$$

Defining $a=\alpha_{\mathrm{good}}-\alpha_{0}$, this expression simplifies to

(11) $$\alpha \left(\tilde{W}\right)=\alpha_{0}+a\;P_{\mathrm{good}}\left(\tilde{W}\right).$$

To describe the width dependence of $P_{\mathrm{good}}\left(\tilde{W}\right)$, we assume that the relative kinetic accessibility of the two pathways varies approximately linearly with normalized trench width. This assumption is expressed through a linear dependence of the log-odds,

(12) $$\ln\;\left(\frac{P_{\mathrm{good}}}{1-P_{\mathrm{good}}}\right)=\frac{{\tilde{W}}_{0}-\tilde{W}}{\tilde{w}},$$

Here Equation (12) is intended as a minimal, physically motivated empirical ansatz rather than a first-principles derivation. More generally, the log-odds $\Delta \left(\tilde{W}\right)\equiv \ln\;\left(P_{\mathrm{good}}/\left(1-P_{\mathrm{good}}\right)\right)$ is expected to be a smooth, monotonically decreasing function of $\tilde{W}$, because increasing trench width simultaneously increases the distance (and thus time) required for the wall-guided front to span the trench while also increasing the interior region available for bulk-like nucleation. In the narrow crossover window, it is therefore natural to retain only the leading (linear) term in a local expansion of $\Delta (\tilde{W})$ about its zero crossing, which motivates Equation (12) and leads to the logistic form in Equation (13). The excellent fits across all parameter sweeps provide strong empirical support for this minimal form. Accordingly, Equation (12) implies the logistic form,

(13) $$P_{\mathrm{good}}\left(\tilde{W}\right)=\frac{1}{1+\exp\;\left(\frac{\tilde{W}-{\tilde{W}}_{0}}{\tilde{w}}\right)}.$$

Substituting this relation yields

(14) $$\alpha \left(\tilde{W}\right)=\alpha_{0}+\frac{a}{1+\exp\;\left(\frac{\tilde{W}-{\tilde{W}}_{0}}{\tilde{w}}\right)}.$$

Here ${\tilde{W}}_{0}$ is defined by $\alpha \left({\tilde{W}}_{0}\right)=\alpha_{0}+a/2$, the midpoint of the sigmoidal alignment–width relation, representing a crossover width separating two competing ordering pathways. For $\tilde{W}<{\tilde{W}}_{0}$, wall-guided propagation typically dominates, leading to a globally aligned state, whereas for $\tilde{W}>{\tilde{W}}_{0}$ bulk-like interior nucleation tends to occur first, resulting in a misoriented, kinetically trapped morphology. Physically, ${\tilde{W}}_{0}$ marks the width at which wall-initiated ordering and interior (bulk-like) nucleation become comparable in kinetic accessibility; equivalently, it corresponds to the condition $\tau_{\mathrm{wall}}\left({\tilde{W}}_{0}\right)∼\tau_{\mathrm{bulk}}\left({\tilde{W}}_{0}\right)$, where $\tau_{\mathrm{wall}}$ is the characteristic time for a wall-guided ordering front to span the trench and $\tau_{\mathrm{bulk}}$ is the waiting time for the first interior nucleation event. The parameter $\tilde{w}$ quantifies the sharpness of this crossover, as reflected in the slope

(15) $${\left.\frac{d\alpha }{d\tilde{W}}\right|}_{\tilde{W}={\tilde{W}}_{0}}=-\frac{a}{4\tilde{w}}.$$

The parameter $\alpha_{0}$ captures the residual anisotropy in the wide-trench limit, reflecting partial ordering that persists near the trench sidewalls after global alignment has broken down in the interior. The amplitude *a* sets the contrast between the wall-dominated aligned state and this wide-trench residual state, such that $\alpha_{\mathrm{aligned}}=\alpha_{0}+a$ denotes the effective alignment degree in the narrow-trench regime. In this framework, ${\tilde{W}}_{0}$ and $\tilde{w}$ control the location and sharpness of the width-driven crossover, respectively, while $\alpha_{0}$ and *a* characterize the asymptotic alignment levels on either side of the transition. Because $\tau_{\mathrm{bulk}}$ depends on the fluctuation level that seeds interior grains (here controlled by the initial disorder amplitude η), the extracted ${\tilde{W}}_{0}$ should be interpreted as an effective crossover descriptor for a given simulation protocol rather than a universal constant. A sensitivity check over η confirms that the same logistic form remains applicable while ${\tilde{W}}_{0}$ shifts systematically (see Figure S2 in the Supporting Information).

**Figure 3:**
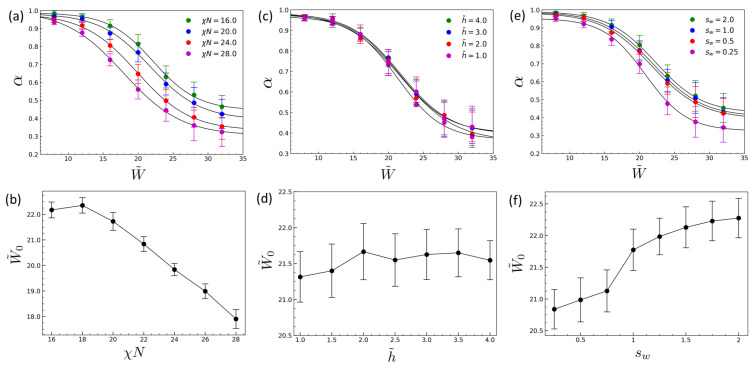
Dependence of the width-driven crossover on molecular and process parameters. Top panels (**a**,**c**,**e**): alignment degree α as a function of normalized trench width $\tilde{W}=W/\lambda$ for (**a**) varying incompatibility χN, (**c**) varying normalized film thickness $\tilde{h}=h/\lambda$, and (**e**) varying sidewall surface field $s_{w}$ (symbols: simulation; solid lines: fits to Equation (10)). Bottom panels (**b**,**d**,**f**): corresponding crossover center ${\tilde{W}}_{0}$ extracted from the fits as a function of (**b**) χN, (**d**) $\tilde{h}$, and (**f**) $s_{w}$. Note that the vertical axes in (**b**,**d**,**f**) are magnified to highlight relatively small but systematic shifts in ${\tilde{W}}_{0}$.

Figure 3 examines how the width-driven crossover responds to changes in key molecular and processing parameters by varying (i) the degree of incompatibility χN (Figure 3a,b), (ii) the normalized film thickness $\tilde{h}=h/\lambda$ (Figure 3c,d), and (iii) the sidewall surface field $s_{w}$ (Figure 3e,f), while holding all other parameters at the baseline condition used in Figure 1. In Figure 3, α is plotted against the normalized width $\tilde{W}=W/\lambda$, and each curve represents a width sweep for a fixed parameter set rather than a single fixed trench width. The common high-alignment plateau at small $\tilde{W}$ reflects a wall-dominated regime shared by all parameter sets, while the parameter dependence is quantified by the shift in the crossover center ${\tilde{W}}_{0}$ extracted from the sigmoidal fits (bottom panels). For all parameter sweeps in which wall-guided ordering persists across the trench, the alignment curves $\alpha (\tilde{W})$ preserve the same sigmoidal dependence on the normalized trench width and are well described by Equation (14). Over the parameter ranges considered here, the transition sharpness remains confined to a narrow interval (typically $\tilde{w}∼2.0$–2.5), whereas the crossover center ${\tilde{W}}_{0}$ shifts substantially. This separation of sensitivities indicates that parameter variations primarily translate the crossover location rather than broadening the transition, as reflected by the small ratio $\tilde{w}/{\tilde{W}}_{0}∼0.1$, thereby establishing ${\tilde{W}}_{0}$ as the dominant descriptor of alignment breakdown.

Figure 3a,b show that increasing the degree of incompatibility χN shifts the crossover to smaller normalized widths, as reflected by a monotonic decrease in ${\tilde{W}}_{0}$. This trend indicates that stronger segregation reduces the maximum width over which wall-guided ordering can propagate before interior nucleation intervenes, thereby biasing the kinetic competition toward the bulk-like pathway. In contrast, the normalized film thickness $\tilde{h}$ has a negligible effect on the crossover over the range examined here (Figure 3c,d). The extracted ${\tilde{W}}_{0}$ remains approximately constant within uncertainty, indicating no systematic dependence on $\tilde{h}$ in this parameter window. This weak thickness dependence can be rationalized by a simple geometric scaling: increasing *h* increases both the sidewall contact area available to seed wall-guided ordering ($A_{w}∼2hL$, where *L* is the trench length) and the interior volume available for bulk like nucleation (V∼WhL) proportionally, so that their relative weighting scales primarily with $A_{w}/V∼2/W$ and is approximately independent of *h* once the morphology is effectively columnar across the thickness. The above geometric cancellation ($A_{w}/V∼2/W$) is expected to hold when the lamellar morphology is effectively columnar across the film thickness (weak *z*-dependence), as in the present simulations with neutral substrate and free surface ($s_{s}=s_{t}=0$) over the thickness window studied in Figure 3c,d. In ultrathin films and/or when the substrate or free surface is selective, vertical commensurability and wetting-induced layering can introduce significant vertical frustration and *z*-dependent morphologies, in which case a stronger thickness dependence of the crossover center ${\tilde{W}}_{0}$ may arise. In the timescale picture, this cancellation implies that increasing *h* does not strongly alter the ratio of the wall-front spanning time to the effective waiting time for an interior nucleation event, so the condition $\tau_{\mathrm{wall}}\left({\tilde{W}}_{0}\right)∼\tau_{\mathrm{bulk}}\left({\tilde{W}}_{0}\right)$ is reached at nearly the same ${\tilde{W}}_{0}$ across the thickness range studied. Here, *h* represents the effective trench depth because the film fills the cavity up to the flat opening plane. Over the thickness range studied ($\tilde{h}=h/\lambda =1$–4), changing *h* primarily rescales both the sidewall contact area and the interior volume proportionally, so the crossover width ${\tilde{W}}_{0}$ depends only weakly on $\tilde{h}$ (Figure 3c,d). Stronger thickness effects reported elsewhere typically involve ultrathin or thickness-modulated films, where vertical confinement/wetting or nonuniform filling can change the ordering pathway [11,37].

On the other hand, the sidewall surface field $s_{w}$ exerts a qualitatively distinct influence (Figure 3e,f). Here $s_{w}$ denotes the (dimensionless) sidewall surface field in Equation (4): $s_{w}=0$ corresponds to neutral sidewalls, and increasing $|s_{w}|$ strengthens preferential wetting and thus accelerates wall-guided ordering. We use $s_{w}=0.5$ as a representative moderate-selectivity baseline. Increasing $s_{w}$ leads to a pronounced increase in ${\tilde{W}}_{0}$, extending the width range over which wall-guided alignment remains kinetically accessible. This dependence clearly saturates at larger $s_{w}$, implying diminishing returns once a sufficiently strong sidewall bias is established. In this regime, strengthening the sidewall bias no longer improves alignment by accelerating wall-guided ordering, and the loss of alignment is instead determined by the onset of bulk-like nucleation in the trench interior.

The contrasting roles of χN, $\tilde{h}$, and $s_{w}$ clarify how material parameters and surface interactions enter the width-driven crossover. Whereas χN and $s_{w}$ primarily bias the competition between wall-guided propagation and interior nucleation, film thickness plays only a secondary role beyond the thin-film limit. The persistence of a common sigmoidal dependence across these parameter sweeps indicates that alignment breakdown is governed by a kinetic competition between wall-guided propagation and bulk-like interior nucleation, rather than by a gradual and continuously tunable loss of order. In this framework, ${\tilde{W}}_{0}$ serves as a kinetic crossover width separating regimes dominated by wall-guided ordering ($\tilde{W}\ll {\tilde{W}}_{0}$) and by interior nucleation followed by domain impingement ($\tilde{W}\gg {\tilde{W}}_{0}$). This timescale-based interpretation also clarifies why parameter variations primarily translate the crossover location rather than altering its sharpness. Parameters that effectively shorten $\tau_{\mathrm{bulk}}$ shift the crossover to smaller $\tilde{W}$, as observed when increasing χN, whereas parameters that accelerate wall-guided ordering (reducing $\tau_{\mathrm{wall}}$) shift the crossover to larger $\tilde{W}$, as observed when increasing $s_{w}$ until saturation is reached. By contrast, once the film is sufficiently thick to support the relevant lamellar ordering modes, variations in $\tilde{h}$ have little effect on either timescale, consistent with the weak thickness dependence of both ${\tilde{W}}_{0}$ and $\tilde{w}$.

While the preceding analysis assumes perfectly vertical sidewalls, practical topographic patterns fabricated by etching commonly exhibit finite sidewall taper, yielding trapezoidal rather than rectangular trench cross-sections. Even modest deviations from $\theta =0^{\circ }$ can modify the lateral confinement as a function of height and alter the geometry of the wall-guided ordering front. Because alignment breakdown in wide trenches is governed by the competition between wall-guided propagation and interior nucleation, such geometric non-idealities can shift the width range over which robust alignment is attainable. Motivated by these considerations, we systematically examine the effect of the sidewall tilt angle θ on the resulting morphologies and alignment metrics under otherwise identical conditions.

**Figure 4:**
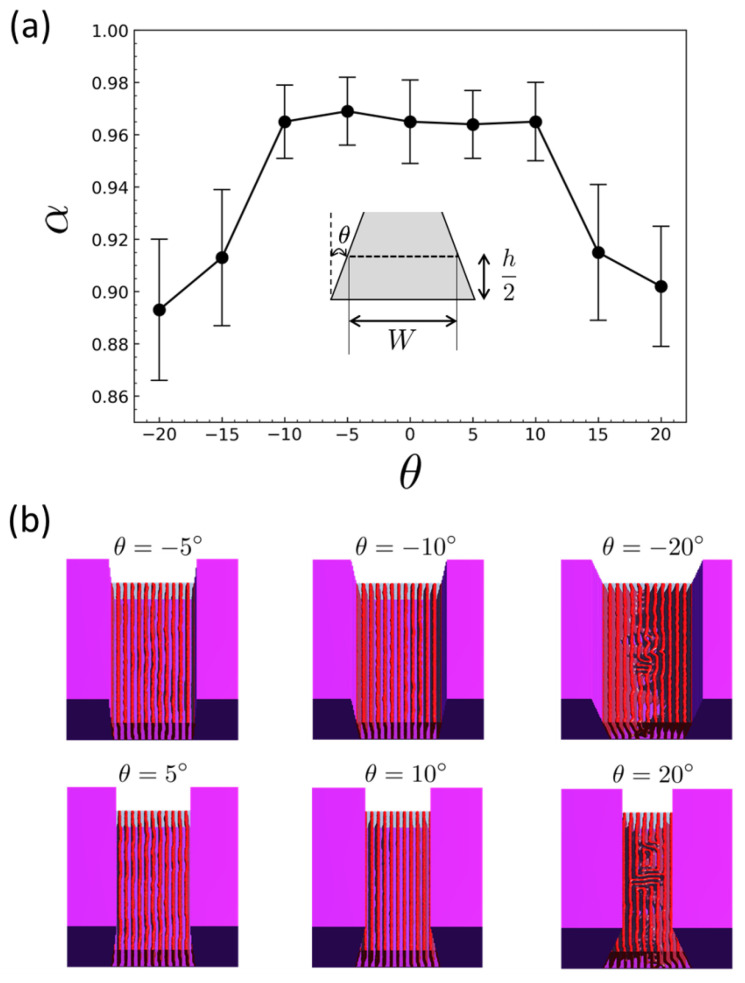
Effect of sidewall tilt (trapezoidal trench cross-section) on lamellar alignment at a fixed nominal normalized width $\tilde{W}=W/\lambda =12$ under the baseline condition of Figure 1 (χN=20 with N=100, $\tilde{h}=h/\lambda =4$, $s_{w}=0.5$, and neutral surface fields $s_{s}=0.0$ and $s_{t}=0.0$). (**a**) Alignment metric α as a function of sidewall tilt angle θ; the inset defines θ (relative to the vertical) and the nominal trench width *W*. Error bars indicate run-to-run variability over independent initial conditions. (**b**) Representative late-time morphologies at selected tilt angles $\theta =-5^{\circ }$, $-10^{\circ }$, $-20^{\circ }$, $+5^{\circ }$, $+10^{\circ }$, and $+20^{\circ }$.

Figure 4 presents the effect of finite sidewall tilt on lamellar alignment in trapezoidal trenches at a fixed nominal width $\tilde{W}=12$, with all remaining parameters held at the baseline condition of Figure 1. Figure 4a quantifies the alignment response using the same Fourier-based metric α employed in the width-sweep analysis, and representative morphologies at selected angles are shown in Figure 4b. Across a broad range of moderate tilts, the alignment remains high: α exhibits a pronounced plateau for $\left|\theta \right|≲10^{\circ }$, with values close to those obtained for the vertical-wall case. In this regime, the corresponding morphologies in Figure 4b show well-registered, trench-parallel lamellae spanning the film, with only minor local distortions. In contrast, for larger tilts ($\left|\theta \right|≳15^{\circ }$–$20^{\circ }$), α decreases appreciably and the morphologies more frequently display lamellar bending and meandering defects in the trench interior, indicating that strong taper introduces geometric frustration via depth-dependent lateral confinement.

A plausible origin of the degradation at large |θ| is the depth-dependent lateral confinement inherent to a trapezoidal trench. We take *z* as the vertical coordinate measured from the substrate plane (z=0) to the film free surface (z=h). As defined in the inset of Figure 4a, the nominal trench width *W* is taken as the width at the mid-height plane, i.e., $W\equiv W_{\mathrm{mid}}=W\left(z=h/2\right)$, which is held fixed as θ is varied. For a constant sidewall tilt angle θ (measured relative to the vertical; θ>0 corresponds to an inward taper so that the trench narrows with increasing *z*), the local trench width varies linearly with height as

(16) $$W\left(z\right)=W-2\left(z-\frac{h}{2}\right)\tan\theta .$$

The corresponding bottom and top widths are then

(17) $$W_{\mathrm{bot}}\equiv W\left(0\right)=W+h\tan\theta ,\;W_{\mathrm{top}}\equiv W\left(h\right)=W-h\tan\theta ,$$

and the total width variation across the film thickness is

(18) $$\Delta W\equiv |W_{\mathrm{top}}-W_{\mathrm{bot}}|=2h|\tan\theta |.$$

Introducing the normalized local width $\tilde{W}\left(z\right)=W\left(z\right)/\lambda$, the corresponding variation in normalized width is

(19) $$\Delta \tilde{W}\equiv \left|\tilde{W}\left(h\right)-\tilde{W}\left(0\right)\right|=\frac{\Delta W}{\lambda }=2\tilde{h}\left|\tan\theta \right|,$$

where $\tilde{h}=h/\lambda$. When $\Delta \tilde{W}∼1$, i.e., when the trench width varies by approximately one lamellar period across the film thickness, a single integer number of lamellae cannot remain simultaneously commensurate throughout the film height without incurring substantial elastic distortion. The system then accommodates the accumulated misfit through lamellar bending and/or defect formation (dislocation-like events), leading to the observed meandering morphologies and a reduction in α at large |θ|. For the present condition $\tilde{h}=4$, $\left|\theta \right|\approx 10^{\circ }$ yields $\Delta \tilde{W}\approx 1.4$, which is consistent with the confinement scale (∼1.4 in normalized units) at which layer-addition transitions occur in strong-segregation analyses of lamellae under one-dimensional confinement [38].

## 4. Concluding Remarks

In this work, we used mesoscale simulations to elucidate how lamellar block copolymer alignment in graphoepitaxial trenches depends on trench geometry and key material/surface parameters. A Fourier-based alignment metric reveals a sharp, sigmoidal decay of alignment with normalized trench width, indicating a width-driven crossover between globally aligned states and misoriented, kinetically trapped outcomes. Time-evolution analysis supports a kinetic picture in which this crossover reflects competition between wall-guided propagation and bulk-like interior nucleation followed by domain impingement. Across parameter sweeps, the dominant sensitivity appears primarily as a horizontal shift in the crossover center ${\tilde{W}}_{0}$, with only modest changes in transition sharpness $\tilde{w}$, establishing ${\tilde{W}}_{0}$ as a compact metric for the characteristic width scale associated with alignment breakdown under a given set of conditions.

Relative to prior SCFT, phase-field, and CDS studies of confined block copolymer ordering that primarily map commensurability windows and/or steady-state morphologies [18,19,20], the present work targets the wide-trench regime where graphoepitaxial alignment commonly fails and casts the breakdown as a kinetic crossover governed by competition between a wall-guided ordering front and bulk-like interior nucleation. This crossover is quantified using a Fourier-based alignment metric together with a compact sigmoidal description, yielding the parameters ${\tilde{W}}_{0}$ and $\tilde{w}$ that directly characterize the accessible alignment window and its systematic shifts with material, surface, and geometric parameters. Extending the same framework to trapezoidal trenches further translates sidewall taper into an explicit tolerance criterion based on the width variation across the film thickness.

From a practical design standpoint, the fitted sigmoidal relation $\alpha (\tilde{W})$ enables a direct estimate of the maximum allowable trench width for a required alignment level $\alpha^{*}$. Once $\left(\alpha_{0},a,{\tilde{W}}_{0},\tilde{w}\right)$ are determined for a given material and processing condition, the corresponding width can be obtained in closed form by inverting Equation (14), without additional simulations.

To extend alignment to wider trenches, the kinetic balance must be shifted in favor of wall-guided propagation, either by strengthening sidewall templating (up to the observed saturation) and/or by selecting conditions that suppress or delay interior nucleation. Within the parameter ranges examined here, increasing incompatibility χN shifts ${\tilde{W}}_{0}$ to smaller widths, whereas increasing the sidewall surface field $s_{w}$ increases ${\tilde{W}}_{0}$ with diminishing returns at large fields; the normalized film thickness exhibits negligible influence on ${\tilde{W}}_{0}$ over the range examined.

The trapezoidal-trench study indicates that high alignment persists for moderate sidewall taper, demonstrating robustness to process-relevant deviations from perfectly vertical sidewalls. A simple geometric criterion follows from the confinement gradient across the film thickness: the normalized width variation satisfies $\Delta \tilde{W}≃2\tilde{h}\left|\tan\theta \right|$, where $\tilde{h}=h/\lambda$. Substantial degradation is expected when $\Delta \tilde{W}$ approaches a critical value $\Delta {\tilde{W}}_{c}≃1.4$ [38]. Setting $\Delta \tilde{W}=\Delta {\tilde{W}}_{c}$ yields an approximate tilt tolerance,

(20) $$|\theta_{c}|\approx \arctan\;\left(\frac{\Delta {\tilde{W}}_{c}}{2\tilde{h}}\right),$$

which captures the observed onset of meandering and the reduction in α when the trapezoidal confinement variation across the film thickness approaches the commensurability-driven instability scale.

Looking forward, the present kinetic-competition framework suggests concrete routes to mitigate alignment loss in the wide-trench regime by targeting the relative timescales $\tau_{\mathrm{wall}}$ and $\tau_{\mathrm{bulk}}$. Promising strategies include (i) accelerating wall-guided ordering fronts through optimized sidewall chemistry and interfacial bias, and (ii) reducing the likelihood of misoriented interior grains by converting homogeneous interior nucleation into guided heterogeneous nucleation via sparse internal seeding features (e.g., weak topographic ribs/guideposts or sparse chemical cues) that do not require dense pre-patterning [39]. In addition, spatially programmed annealing protocols (e.g., zone annealing [40,41]) may offer a practical pathway to bias ordering toward a single orientation by controlling the ordering history. These directions provide a rational basis for expanding the process window of wide-trench graphoepitaxy through geometry- and kinetics-aware design.

## Data Availability

The data presented in this study are available on request from the corresponding author.

## Supplementary Materials

The following supporting information can be downloaded at: https://www.mdpi.com/article/10.3390/polym18050557/s1, Figure S1: Additional Sensitivity Test with Respect to Degree of Polymerization. Sensitivity of the width-driven crossover to *N* at fixed chiN and normalized geometry; Figure S2: Fluctuation-Level Dependence of the Width-Driven Crossover. Effect of initial fluctuation amplitude on the extracted crossover width.
