# Supplementary material for: Graphoepitaxial Control of Lamellar Block Copolymer Alignment in Wide Trenches: Effects of Trench Width and Sidewall Tilt
Source: Polymers (Basel). 2026 Feb 25;18(5):557. doi: 10.3390/polym18050557 (PMC12987211; doi:10.3390/polym18050557)
Supplement: Supplementary file 1 [file polymers-18-00557-s001.zip › polymers-4144756-supplementary.pdf]

Supporting Information

Graphoepitaxial Control of Lamellar Block Copolymer Alignment in Wide Trenches: Effects of Trench Width and Sidewall Tilt

June Huh
Department of Chemical and Biological Engineering,
Korea University, Seoul 02841, Korea
junehuh@korea.ac.kr

Additional Sensitivity Test with Respect to Degree of Polymerization

To assess the sensitivity of the graphoepitaxial alignment behavior to the degree of polymerization N at fixed segregation strength ($\chi N = 20$), we performed additional simulations at $N = 200$ and 300 in addition to the baseline case $N = 100$.

For each N, all N-dependent coefficients were recomputed according to Eq. (3) of the main text. The confinement geometry was defined in normalized form and kept identical across cases, such that $\tilde{W} \equiv W/\lambda = 21$, $\tilde{L}_y \equiv L_y/\lambda = 30$, and $\tilde{h} \equiv h/\lambda$ were fixed. Accordingly, L_y was adjusted for each N to maintain $\tilde{L}_y = 30$, and all lengths were scaled by the intrinsic lamellar period λ corresponding to that parameter set.

Figure S1 shows representative late-time morphologies for $N = 100$, 200, and 300. The alignment metric α (defined in Eqs. (6)–(9) of the main text) remains nearly unchanged across this range ($\alpha = 0.705$, 0.697, and 0.713, respectively), indicating no systematic trend with N under identical normalized confinement conditions.

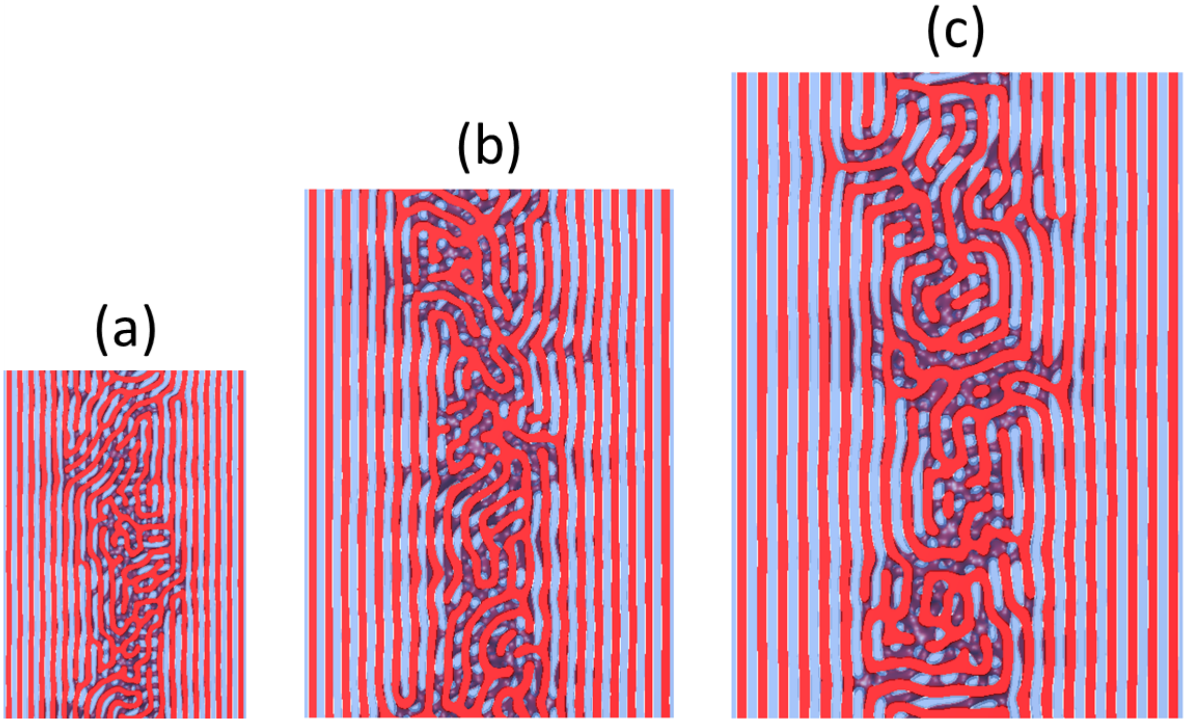

Figure 1: Sensitivity of graphoepitaxial alignment to the degree of polymerization N at fixed $\chi N = 20$ under identical normalized geometry ($\tilde{W} = 21$, $\tilde{L}_y = 30$, and fixed $\tilde{h}$). Panels (a)–(c) correspond to $N = 100$, 200, and 300, respectively. All N-dependent coefficients were recomputed according to Eq. (3) of the main text.

Fluctuation-Level Dependence of the Width-Driven Crossover

In the present CDS simulations, no additional time-dependent noise is applied during the evolution; stochasticity is introduced solely through the disordered initial condition $\Psi(\mathbf{r}, 0) \sim u(-\eta, \eta)$ (uniform distribution). To examine how the fluctuation level affects the width-driven kinetic crossover, we performed additional width sweeps using a reduced initial disorder amplitude ($\eta = 0.001$) in comparison to the baseline case ($\eta = 0.1$).

All other parameters were kept identical to the representative condition of Fig. 1 in the main text ($\chi N = 20$, $N = 100$, $\tilde{h} = 4$, $s_w = 0.5$, $s_s = s_t = 0$), and alignment values were obtained by averaging over multiple independent runs for each $\tilde{W}$.

Figure S2 shows the resulting alignment degree $\alpha(\tilde{W})$ together with fits to Eq. (14) of the main text. The sigmoidal (logistic) form provides an excellent description in both cases. Reducing η systematically shifts the fitted crossover center from $\tilde{W}_0 = 21.9$ ($\eta = 0.1$) to $\tilde{W}_0 = 22.8$ ($\eta = 0.001$), consistent with delayed fluctuation-seeded interior grain formation at lower fluctuation levels. These results indicate that while the crossover location depends on the fluctuation level through its influence on τ_{bulk}, the logistic description remains applicable. Accordingly, $\tilde{W}_0$ should be interpreted as an effective descriptor of the accessible alignment window for a given simulation protocol (including fluctuation amplitude), rather than as a universal constant.

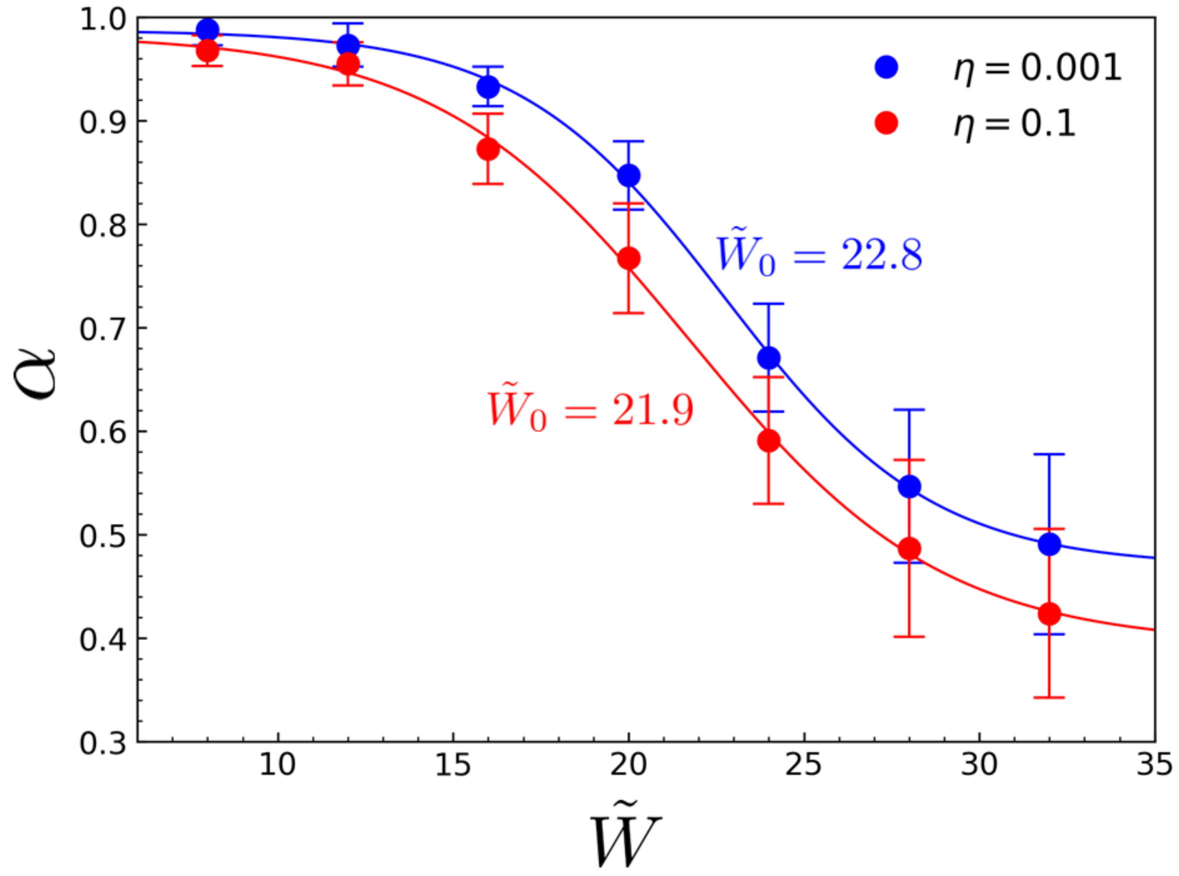

Figure 2: Sensitivity of the width-driven crossover to the initial disorder amplitude η. Alignment degree α as a function of normalized trench width $\tilde{W}$ for $\eta = 0.1$ (red) and $\eta = 0.001$ (blue) under otherwise identical conditions. Solid lines are fits to Eq. (14) of the main text. While the sigmoidal form remains an excellent description in both cases, the fitted crossover center shifts from $\tilde{W}_0 = 21.9$ to $\tilde{W}_0 = 22.8$ as η decreases, consistent with delayed interior grain formation at lower fluctuation levels.
